# Feasibility of using ultra‐low pulse rate fluoroscopy during routine diagnostic coronary angiography

**DOI:** 10.1002/jmrs.293

**Published:** 2018-07-16

**Authors:** Mohamed Khaldoun Badawy, Matthew Scott, Omar Farouque, Mark Horrigan, David J. Clark, Robert K. Chan

**Affiliations:** ^1^ Monash Imaging Monash Health Clayton Victoria Australia; ^2^ School of Health and Biomedical Sciences RMIT University Bundoora Victoria Australia; ^3^ Cardiovascular Procedure Centre Warringal Private Hospital, Ramsay Healthcare Heidelberg Victoria Australia; ^4^ Department of Cardiology Austin Health Heidelberg Victoria Australia; ^5^ Faculty of Medicine, Dentistry and Health Sciences University of Melbourne Melbourne Victoria Australia

**Keywords:** Fluoroscopy, interventional cardiology, radiation dose optimisation, radiation protection

## Abstract

**Introduction:**

Coronary angiogram, while a powerful diagnostic tool in coronary artery disease, is not without an associated risk from ionising radiation. There are a number of factors that influence the amount of radiation the patient receives during the procedure, some of which are under the control of the operator. One of these is an adjustment of the fluoroscopic pulse rate. This study aims to assess the feasibility of using ultra‐low pulse rate (3 pulses per second(pps)) fluoroscopy during routine diagnostic coronary angiogram procedures and the effect it has on fluoroscopy time, diagnostic clarity and radiation dose.

**Methods:**

A retrospective study of three operators each undertaking 50 coronary angiogram procedures was performed. One of the operators used a pulse rate of 3 pps and 6 pps for fluoroscopic screening while the control groups used the standard 10 pps mode utilised at this centre.

**Results:**

Results demonstrated no reduction of diagnostic clarity, up to a 58% reduction in Dose Area Product and no increase in fluoroscopy time with the 3 pps setting.

**Conclusions:**

Findings from this pilot study suggest that utilisation of ultra‐low pulse rate fluoroscopy in routine transfemoral diagnostic coronary angiography in the catheterisation laboratory is feasible.

## Introduction

The coronary angiogram (CA) is a powerful diagnostic tool to investigate coronary artery disease. The radiation dose associated with a CA procedure is typically 5–10 mSv which is similar to a computed tomography scan.^1^ Radiation exposure may present a risk of carcinogenesis to the patient during their lifetime.^2^ Furthermore, the high radiation dose absorbed by the skin in a large patient or a prolonged procedure introduces the risk of skin tissue reactions, ranging from transient erythema to permanent damage requiring surgical intervention.^3^ Both the stochastic (associated attributable carcinogenesis) and deterministic (tissue reaction severity) effects are related to cumulative radiation dose. Moreover, the X‐rays scattered from the patient expose staff in the catheterisation laboratory to ionising radiation. Previous studies have suggested an occupational risk to staff with potential for development of posterior lens opacities and brain and neck tumours.^4^, ^5^ Consequently, efforts need to be made to keep the radiation dose as low as reasonably achievable during the procedure, which would decrease any potential risk for staff and patients.

Fluoroscopy is necessary to perform a coronary angiogram to guide all aspects of the procedure with a typical pulse rate of 10–15 pulses per second (pps). Also, coronary artery cine acquisitions are typically performed at 10–15 frames per second (fps) to image the moving heart efficiently. Mainly due to the high pulse rates and frame rates during a routine CA procedure, high radiation doses are delivered as compared to other diagnostic X‐ray procedures. Utilising the accepted historical pulse rate standard of 10–15 pps may not be essential for adequate imaging.

Significant dose optimisation is possible by utilising lower pulse rates during fluoroscopy screening. Reduction in pulse rate from the standard 15 to 7.5 pps can result in a significant decrease in radiation dose.^6^, ^7^, ^8^ However, there are no data in the clinical setting which addresses the impact of further reductions in pulse rate below 7.5 pps. A potential disadvantage in using lower fluoroscopic pulse rates relates to the lower temporal resolution with image ghosting and a lack of smooth cadence to the image run. These degrading effects have the potential to impede the procedure and place a limit on minimum pulse rates achievable.

In this study, the ability to use ultra‐low pulse rate fluoroscopy (3 pps) was examined during diagnostic transfemoral CA in an adult population. Assessment of radiation dose and clinical feasibility was done, and the following questions were asked:

1. Does the 3 pps setting provide adequate temporal ‘clarity’ to the operator?
2. Does the 3 pps setting lead to longer fluoroscopy time to compensate for degraded images?
3. Is there a worthwhile net reduction in radiation dose when the 3 pps setting is used?

## Methods

A retrospective study was undertaken on patients requiring routine transfemoral CA in a well‐established cardiac catheterisation laboratory. Three cardiologists participated, all with greater than ten years’ experience in diagnostic and interventional cardiac catheterisation. One cardiologist used a fluoroscopic rate of 3 pps (Operator A1) and 6 pps (Operator A2) while the other two (Operator B and Operator C) used 10 pps. The CA cases were selected from 200 consecutive studies (50 per study group) conducted between February 2014 and August 2015. With the initial acceptance of the unit, Operator A utilised 6 pps settings, and this became standard practice for the operator from February 2014 until September 2014. Following further optimisation of protocols, 3 pps was the standard protocol settings for all CA procedures for Operator A. The two operators who used 10 pps acted as control groups. Two control groups were chosen to lower the probability of falsely detecting a difference based on different operator technique. To ensure patient diagnosis was not compromised at ultra‐low pulse rate settings, higher pulse rate settings were available to Operator A if any difficulties were found during the procedures. None of the 50 consecutive studies required a change in protocol to higher pulse rates.

The angiography system used was a single plane Siemens Artis Q Floor 2014 (Siemens Healthcare, Forchheim, Germany). The modification of the standard noise reduction algorithms and frame averaging functions were required in the 3 pps and 6 pps fluoroscopy protocol to ensure sufficient temporal resolution. This was achieved through the Siemens K‐factor in the modified 3 pps protocol. A K‐factor of Auto5 was selected to remove the ghosting and lag due to the decreased pulse rate. In the standard setting, the K‐factor correction was switched off. All three cardiologists used identical acquisition settings with a cine acquisition frame rate of 10 fps. Standard radiation dose saving measures were used by all operators including low dose rate settings. Primary data collected for each group included total accumulated dose area product (DAP), reference air kerma (RAK), and fluoroscopy time (FT). Secondary data were collected for the number of cine stored acquisitions, vascular access site and patient characteristics including body mass index and gender.

The data were analysed for statistical differences between the DAP, reference air kerma and fluoroscopy time when grouped by the operator using one‐way analysis of variance (ANOVA) with post hoc analysis using Tukey's ‘Honest Significant Difference’ (HSD) method. Data that did not conform to a normal distribution were analysed using the Kruskal–Wallis test with a Bonferroni correction for post hoc analysis. Categorical data were compared using the chi‐square test. The significance threshold was set at 0.05. Statistical analyses were performed using R version 3.3.1 (R Foundation for Statistical Computing, Vienna, Austria) with the Rcmdr package version 2.3‐2.

Quantitatively, image quality was assessed using 20 cm of the CIRS Model 901 phantom compliant with NEMA standard XR21 for cardiovascular fluoroscopic benchmarking. Static image quality and temporal image quality were evaluated on the standard 10 pps and the ultra‐low 3 pps fluoroscopy settings. Image quality assessment was undertaken by a medical physicist and a radiographer. Where agreement was not found, a second radiographer was asked to assess the image quality and the majority decision was recorded. Qualitatively, image quality was evaluated by interviewing Operator A who was using the 3 pps. Operator A was asked if they were able to locate and catheterise the coronary arteries and enter the left ventricle, if they had to revert to the standard 10 pps setting during any procedure, or if they felt that the reduced image quality added an unnecessary burden to the procedure.

This project was subject to Ethics approval by the Research Ethics Committee at the Austin Hospital (LNR/16/Austin/24).

## Results

**Figure 1:**
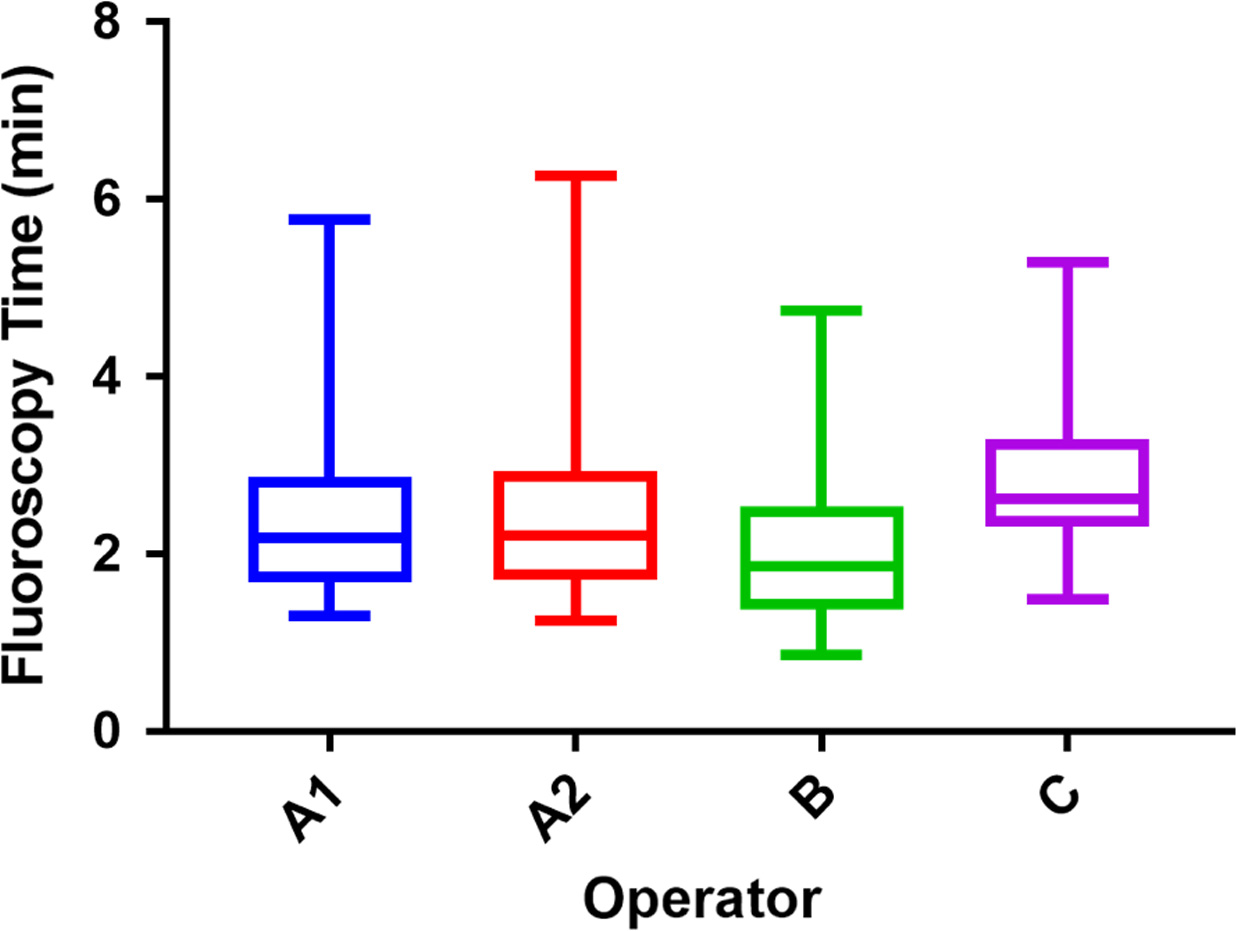
Comparison of fluoroscopy time between operators utilising the 3 pps, 6 pps and 10 pps setting. There is a significant difference between Operator C and all the other study groups.

Patient characteristics are presented in Table 1, and procedural characteristics are presented in Table 2. The difference between the DAP, RAK and FT was compared between four different study groups utilising 3 pps, 6 pps and 10 pps. There was a statistically significant difference in FT values between the different operators (*P* < 0.001) with 2.2 mins for Operator A1, 2.2 mins for Operator A2, 1.9 mins for Operator B and 2.6 for Operator C. A post hoc analysis showed that there was a statistically significant difference for FT between Operator C and all other study groups (*P* < 0.001; Fig. 1). However, no significant difference was detected between Operator A1, A2 and B.

**Table 1:** Patient characteristics in the different study groups

Characteristic	Operator A1 (*n* = 50)	Operator A2 (*n* = 50)	Operator B (*n* = 50)	Operator C (*n* = 50)	*P*‐value
Male, no./total (%)	24/50 (48)	25/50 (50)	20/50 (40)	30/50 (60)	0.3
Age, mean (SD), years	74 (9)	72 (9)	71 (11)	70 (10)	0.2
Height, mean (SD), cm	163 (23)	171 (12)	161 (21)	168 (10)	0.02^a^
Weight, mean (SD), kg	77 (17)	83 (18)	72 (15)	83 (13)	0.0008^b^
Body mass index, mean (SD), kg.m^−2^	28 (4)	28 (6)	27 (4)	29 (4)	0.09

a Stastistical signficance detected between A2 and B, no significant differences detected between the other groups.

b Statistical difference detected between A2 and B and B and C, no significant differences detected between the other groups.

**Table 2:** Procedural characteristics in the different study groups

Characteristic	Operator A1 (*n* = 50)	Operator A2 (*n* = 50)	Operator B (*n* = 50)	Operator C (*n* = 50)	*P*‐value
Left ventriculogram, no./total (%))	43/50 (86)	45/50 (90)	40/50 (80)	48/50 (96)	0.09
Diagnostic acquisitions, mean (SD)	10 (2)	10 (1)	8 (2)	10 (2)	<0.001^a^
Dose area product, median (IQR), Gy.cm^2^	6.34 (4.73–7.94)	9.10 (5.40–12.96)	9.62 (7.95–14.96)	15.02 (9.21–20.53)	<0.001^b^
Reference air kerma, median (IQR), mGy	101 (72–130)	134 (85–206)	168 (126–244)	236 (148–321)	<0.001
Fluoroscopic time, median (IQR), minutes	2.2 (1.7–2.9)	2.2 (1.7–2.9)	1.9 (1.4–2.5)	2.6 (2.3–3.3)	<0.001

a Operator B significantly different to A1, A2, and C, no significant differences detected between the other groups.

b Operator A1 significantly different to A2, B, and C. Operator A2 significantly different to Operator C.

**Figure 2:**
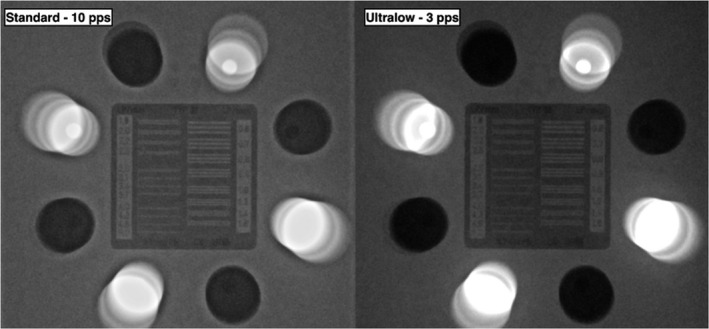
Comparison of the static image quality using the standard 10 pps setting and the modified 3 pps setting and the CIRS Model 901 phantom.

**Figure 3:**
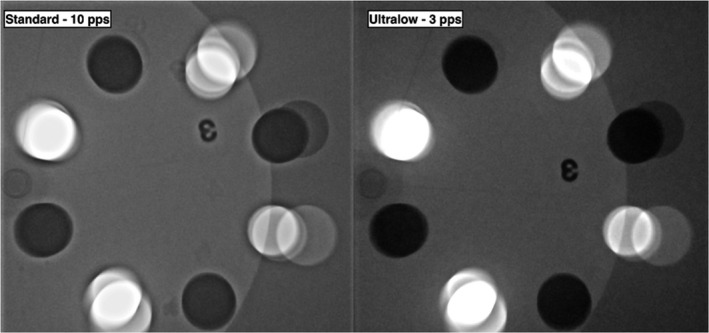
Comparison of the temporal image quality using the standard 10 pps setting and the modified 3 pps setting and the CIRS model 901 phantom. Note the reduction in image ghosting in the modified 3 pps setting through the k‐factor correction.

The difference in quantitative image quality is presented in Table 3. There was a decrease in exposure parameters which resulted in a slight decrease in both perceived resolution and contrast in the modified ultra‐low 3 pps setting (Fig. 2). There was some image ghosting in the standard 10 pps protocol. However, this was not present in the 3 pps protocol (Fig. 3) due to the K‐factor correction. Based on an interview, Operator A was able to accomplish all cases, i.e., locate and catheterise both coronary arteries and enter the left ventricle using 3 pps fluoroscopy to acquire coronary angiograms adequate for clinical diagnostic purpose without deviation from the protocol. It was also noted that a short transition phase was required for the eye to adjust to the lower temporal resolution of fluoroscopic images.

**Table 3:** Image quality assessment of the standard 10 pps fluoroscopic settings versus the 3 pps settings using a NEMA standard XR21 fluoroscopic benchmarking phantom

Groups	10 pps	3 pps
X‐ray parameters		
kVp	77	77
Tube current (mA)	179	166
Dose setting	Low	Low
Added filteration	Cu 0.6	Cu 0.6
Dose rate (uGy/s)	72	21
Static image quality		
Line pairs	2.2	1.6
Iodine group 1	7	6
Iodine group 2	0	0
Iodine group 3	0	0
Iodine group 4	0	0
Air cylinders	2	2
Aluminium cylinders	4	4
X‐ray parameters		
kVp	81	77
Tube current (mA)	96	148
Dose setting	Low	Low
Added filteration	Cu 0.3	Cu 0.6
Dose rate (uGy/s)	80	20
Temporal image quality		
Moving wires	3	3
Dots	2	2
Image ghosting	Yes	No

**Figure 4:**
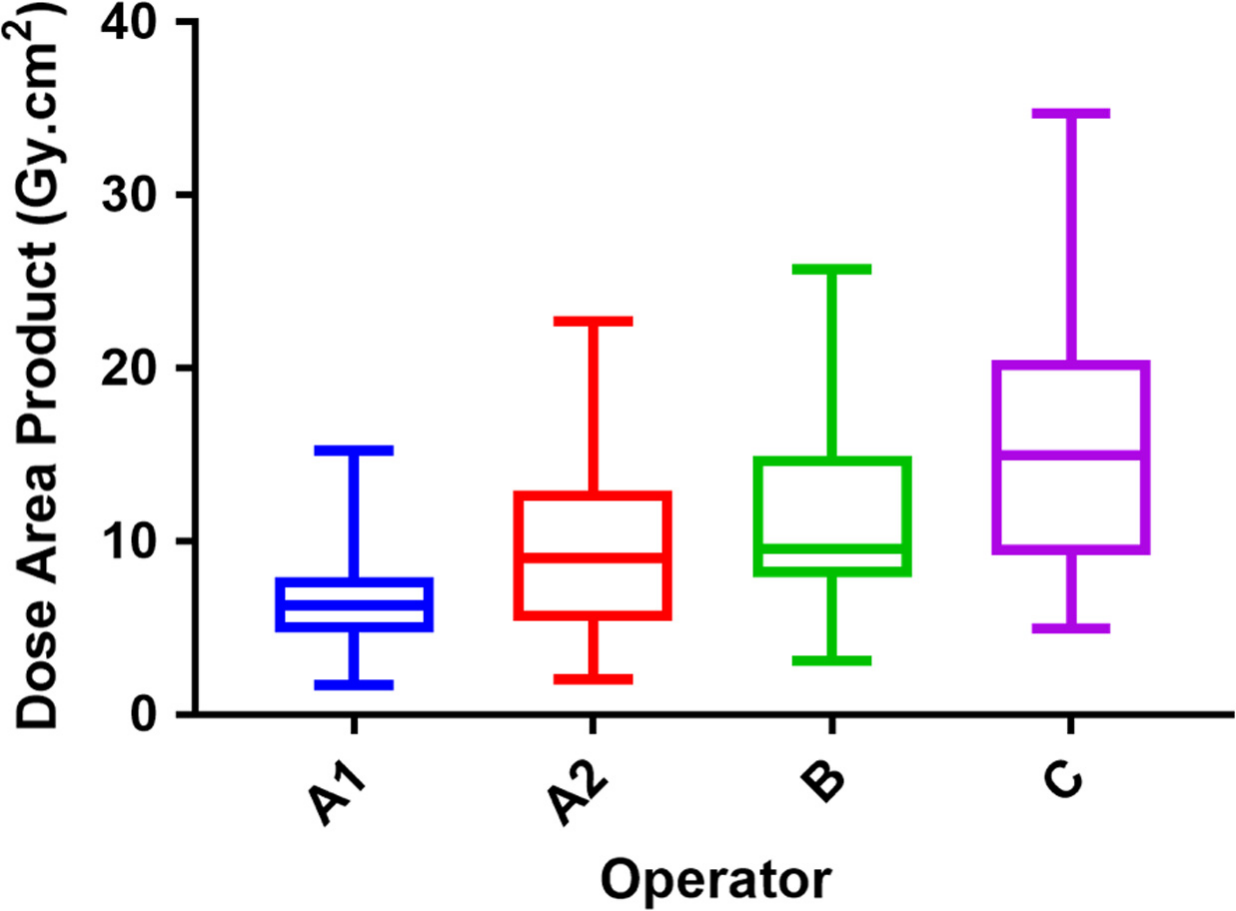
Comparison of DAP values between operators utilising the 3 pps, 6 pps and 10 pps setting. Operator A1 is statistically significantly lower than Operators A2, B and C. Operator A2 is statistically significantly lower than Operator C.

There was a statistically significant difference in DAP values between operators (*P *< 0.001), with a mean rank DAP value of 6.34 Gy.cm^2^ for Operator A1 (3 pps), 9.1 Gy.cm^2^ Operator A2 (6 pps) 9.62 Gy.cm^2^ for Operator B (10 pps) and 15.02 Gy.cm^2^ for Operator C (10 pps). A post hoc analysis showed that there was a statistically significant reduction in DAP between Operator A1 and each of the other groups (*P* < 0.001; Fig. 4). Additionally, there was a statistically significant difference between Operator A2 and Operator C (*P* = 0.004; Fig. 4).

**Figure 5:**
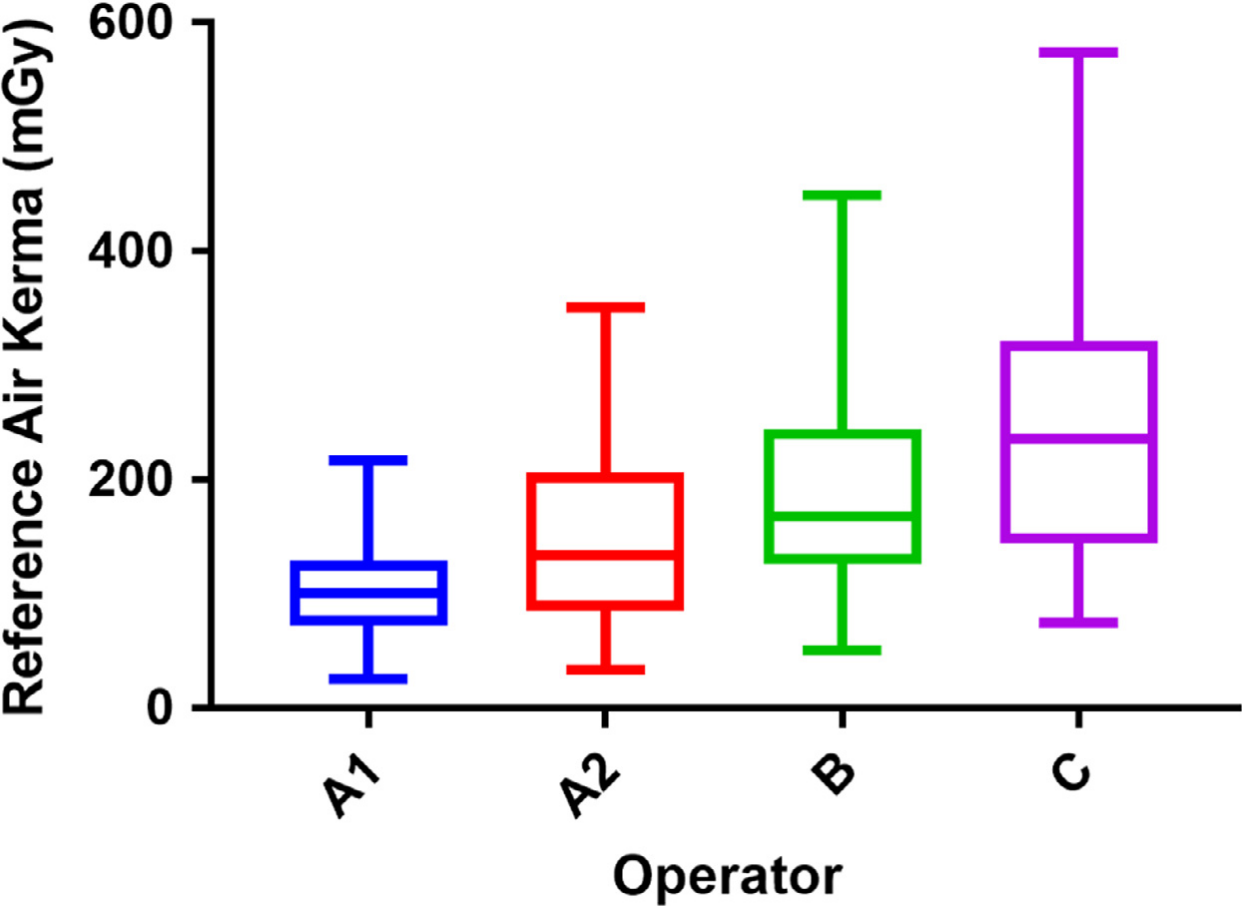
Comparison of reference air kerma values between operators utilising the 3 pps, 6 pps and 10 pps setting. Operator A1 is statistically significantly lower than Operator A2, B and C. Operator A2 is statistically significantly lower than Operator C.

Finally, there was a statistically significant difference in RAK values between the different operators (*P* < 0.001), with a mean rank RAK value of 101 mGy for Operator A1, 133 mGy for Operator A2, 168 mGy for Operator B and 236 mGy for Operator C. A post hoc analysis showed that there was a statistically significant reduction in RAK between Operator A1 and each of the other groups (*P* < 0.001; Fig. 5). Additionally, there was a statistically significant difference between Operator A2 and Operator C (*P* = 0.005; Fig. 5).

## Discussion

The results of this study demonstrated that compromise of imaging clarity during fluoroscopy by utilising ultra‐low pulse rate does not adversely affect the performance of catheterisation by the operator while having a significant impact on radiation dose reduction. Several studies show that the typical radiation dose associated with CA procedures is 14–63 Gy.cm^2^.^1^, ^9^, ^10^ Australian diagnostic reference levels for CA are not published at the time of writing to compare local radiation doses associated with CA; however, a large multicentre study in Australia reported a median DAP of 39.08 Gy.cm^2^.^11^ The median dose for the procedures using ultra‐low pulse rate fluoroscopy in this study was 6.34 Gy.cm^2^.

It is important to note that the primary purpose of the fluoroscopic screening within this study is for the guidance of the catheter and all diagnostic images were taken utilising the standard 10 fps acquisition protocol. The reduction in radiation dose achieved can be partially attributed to the change in fluoroscopic settings. The accumulated procedural radiation dose in the 3 pps group was lower than that of the 10 pps control groups by as much as 58%. The reduction for Operator A when utilising 3 pps as compared to 6 pps is 30%. The only change between Operator A1 and A2 subgroups is the fluoroscopic pulse rates. This indicates that by manipulating only the fluoroscopic dose without adjusting the cine acquisition protocols can in itself lead to notable dose optimisation. Also, our clinical data support a previous phantom study that showed a significant radiation dose reduction when using ultra‐low pulse rates in a simulated environment.^12^ This study also showed that the radiation dose in CA could be optimised to significantly lower ranges than stated in the existing literature.

The dose reduction to the patient will consequently result in a reduction of radiation exposure to the staff members. This effect has been demonstrated by Abdelaal et al. when reducing the frame rate from 15 fps to 7.5 fps.^13^ Therefore, the results of the dose reduction achieved through the decrease in pulse rate in this study can be an effective radiation protection strategy for the catheterisation laboratory staff.

Other clinical studies have used pulse rate, and frame rate manipulation as a method of radiation dose reduction; however, only pulse rates as low as 7 pps have been reported.^6^, ^7^, ^8^ This study showed that it is possible to reduce the pulse rate even further (as low as 3 pps), and consequently further reducing radiation dose, by working with the manufacturer of the fluoroscopic equipment to modify the standard noise reduction algorithms and frame averaging functions. The lower the pulse rate utilised, the higher noise, since fewer X‐ray photons are forming the image, and therefore additional noise suppression is needed. This may not be possible on older systems due to the limitations of the system's software. Thus, the authors recommend that when utilising ultra‐low pulse rates in CA it should be done in consultation with the manufacturer in the initial protocol set up. This will ensure any image ghosting, and perceived cadence is adjusted to allow sufficient image clarity.

There was a measurable decrease in spatial and contrast resolution in the 3 pps setting. The temporal resolution was improved in the 3 pps by adjusting the image processing algorithms. This demonstrates that in conjunction with the manufacturers, the visual image quality at the lower pulse rates can be manipulated to resemble that of the higher pulse rates.

The results of the interview also revealed that there was a short period required to adapt to the altered image quality. However, this was not a significant limitation. In implementing this method of dose optimisation, consideration could be given to lowering the pulse rate from 10 pps to 3 pps in incremental steps. For example, it may be preferable to lower the pulse rate to an intermediary level such as 6 pps which was used by Operator A to assist in the transition to 3 pps.

Another concern of this study was that lowering the pulse rates could result in prolonged FT to compensate for the degraded image quality. The results showed that there was no increase in FT in the low pulse rate group as compared to the control groups.

### Limitations

The first limitation of this study is the ability to assess the data separately as radiation exposure due to fluoroscopic screening and acquisition runs. This would provide the benefit of determining the magnitude of dose reduction as a consequence of the altered fluoroscopic settings only. The addition of Operator A data utilising 6 pps and 3 pps attempts to address this limitation by accounting for the interoperator differences which may alter the overall study dose. However, due to the retrospective nature of this study, access to all the data required to assess fluoroscopic screening individually was unworkable. Future studies of this nature should determine the significant difference in radiation dose between fluoroscopic screening, not the overall study radiation dose.

Second, the qualitative opinions on acceptable image quality in the 3 pps study group were provided only by one operator. Future studies would benefit from testing the ultra‐low fluoroscopic settings on more than one operator to recommend if it is feasible for a broader cardiologist cohort.

## Conclusion

Findings from this pilot study suggest that utilisation of ultra‐low pulse rate fluoroscopy in routine transfemoral diagnostic coronary angiography in the catheterisation laboratory is feasible. Adoption of this protocol may lead to significant reduction in radiation exposure to the patient and laboratory staff without associated complications. The application of this approach in interventional and transradial catheterisation procedures may be suitable for further investigation as it may potentially demonstrate more substantial radiation dose reduction.

## Conflict of Interest

The authors declare no conflict of interest.
